# Mission-based optimal morphing parameters for rotors with
 combined chord and twist morphing

**DOI:** 10.12688/openreseurope.14060.1

**Published:** 2021-10-12

**Authors:** Rohin Kumar Majeti, Stephan Benz

**Affiliations:** 1 German Aerospace Center (DLR), Braunschweig, Germany; 2 Technical University of Braunschweig, Braunschweig, Germany

**Keywords:** SABRE, Morphing, Helicopter rotor, Particle swarm optimization, Optimization

## Abstract

**Background: **The fixed geometry rotor blades in today’s helicopters do not give the best performance throughout the duration of any mission. However, low-speed and high-speed flights have different geometrical requirements for the shape of the most efficient rotor blades. With advancements in morphing technologies, these can be applied to change the shape of the blades in different flight regimes.

**Methods: **Two different helicopter rotor morphing concepts – namely, the linearly variable chord extension and the torque-tube based twist - under the framework of the European project SABRE were investigated for their optimal geometric parameters using a Particle Swarm Optimization (PSO) algorithm. Since the morphing parameters were dependent on the mission profile, three different missions representing typical helicopter applications were chosen. The optimization problem was posed both as single objective (power) and as multi-objective (power, tip elastic torsion and vibratory hub load). Based on the insights drawn from these investigations, a rotor was set up including both morphing concepts in a single blade.

**Results: **The rotor with combined chord and twist morphing was shown to have better performance than the baseline blade, while keeping the penalty on the elastic torsion and vibration of the rotor to a minimum.

**Conclusions: **Chord and twist are both important parameters determining the efficiency of a rotor blade. Since they have non-overlapping requirements, combining the two morphing concepts into a single blade can yield higher performance than the individual ones.

## Plain language summary

In order to have the best performance, helicopters require their rotor blades to have different shapes in hover and at various flight speeds. However, due to manufacturing constraints and technology limitations, most helicopter rotor blades have rectangular shapes as a compromise. As technology progresses, it is possible today to have morphing blades which change shape with change in flight speed. Using an optimization algorithm called particle swarm optimization (PSO), the current work aims to find the best possible rotor blade shape during the course of a mission. Two different morphing concepts - one involving linearly variable chord along the length of the blade and another with varying twist along the length of the blade, are investigated. Based on the insights drawn from this study, a blade having varying chord (width of rotor blade) in the inboard sections of the blade and varying twist (angle that the chord line at any section makes with the chord line at the root section) in the outboard sections of the blade was set up. This blade with combined morphing characteristics was shown to have better performance than the baseline blade.

## Introduction ^1^

It is a challenge to design a helicopter’s main rotor to operate efficiently through all stages of a mission while maintaining its vibration and stress limits. The fixed geometry rotor in current helicopters is a compromise between various competing needs of different flight regimes in a mission. Rather, a morphing rotor capable of changing its geometry can handle these competing objectives better. With the primary objective of reducing helicopter emissions, several rotor morphing concepts are being researched within the European project Shape Adaptive Blades for Rotorcraft Efficiency, SABRE www.sabreproject.eu ^1^. Within SABRE, the German Aerospace Center (DLR) is responsible for the variable chord extension concept ^2^ while twist morphing is the focus of research for a few other partners ^3, 4^.

Optimization techniques have been used in helicopter rotor design for more than three decades now ^5^. These techniques reduce the workload of obtaining the optimum design from the designer. Since analysis and design of the helicopter rotor is multidisciplinary in nature, over the years, researchers have performed optimization by taking into account the couplings between aerodynamics, dynamics and structures ^6– 8^. These optimization procedures have dealt with a large number of design variables and multiple objectives like performance, vibration, noise and weight being met simultaneously. Optimization of morphing airfoils for helicopter rotor blades have been dealt with, recently, in 9.

Several strategies for optimization have been discussed at length in 10. Many of these strategies require gradient information to determine the direction of the global optimum. However, these strategies are met with problems when there are multiple local minima. Evolutionary methods of optimization like genetic algorithm, on the other hand, require only the functional values and not the gradients. Particle swarm optimization (PSO) is an evolutionary algorithm based on the behavior of a colony of living organisms such as a flock of birds ^11^. The method does not require gradients and can find global optima based on randomized start points and the knowledge of the current global and personal minima through its iterative, population-based character. This makes it suitable for running iterative and computationally expensive numeric programs. PSO has recently gained popularity for use in helicopter rotor design ^12– 14^. 

The geometry of a morphed rotor, whether morphed in chord or twist, is described by a set of additional parameters. Since requirements for rotor design, like performance or vibrations, are different for hover and for forward flight, the geometry of the morphed rotor changes throughout any particular mission. Thus, morphing parameters are mission-dependent, and also change during the course of the mission. In this work, the optimal geometrical parameters for a morphing rotor were determined and presented using an established optimization algorithm. Different standard missions were investigated for their effect on the morphing parameters. The combined effect of the investigated systems in a single blade was also looked into.

## Methods

### Morphing concepts

Two morphing concepts will be considered in this paper – the linearly variable chord-extension morphing concept of DLR ^2^ and the twist morphing using shape memory alloy (SMA) torque-tubes concept of CIRA Italy ^4^.

**Figure 1.**
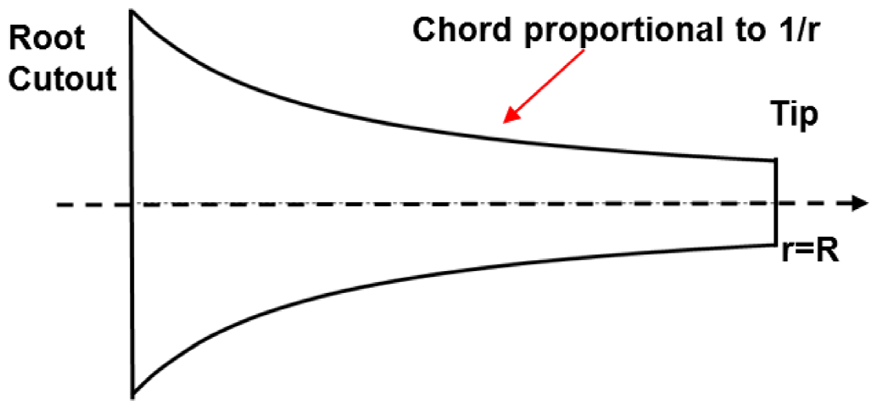
Top view of optimum hovering rotor blade.

**Figure 2.**
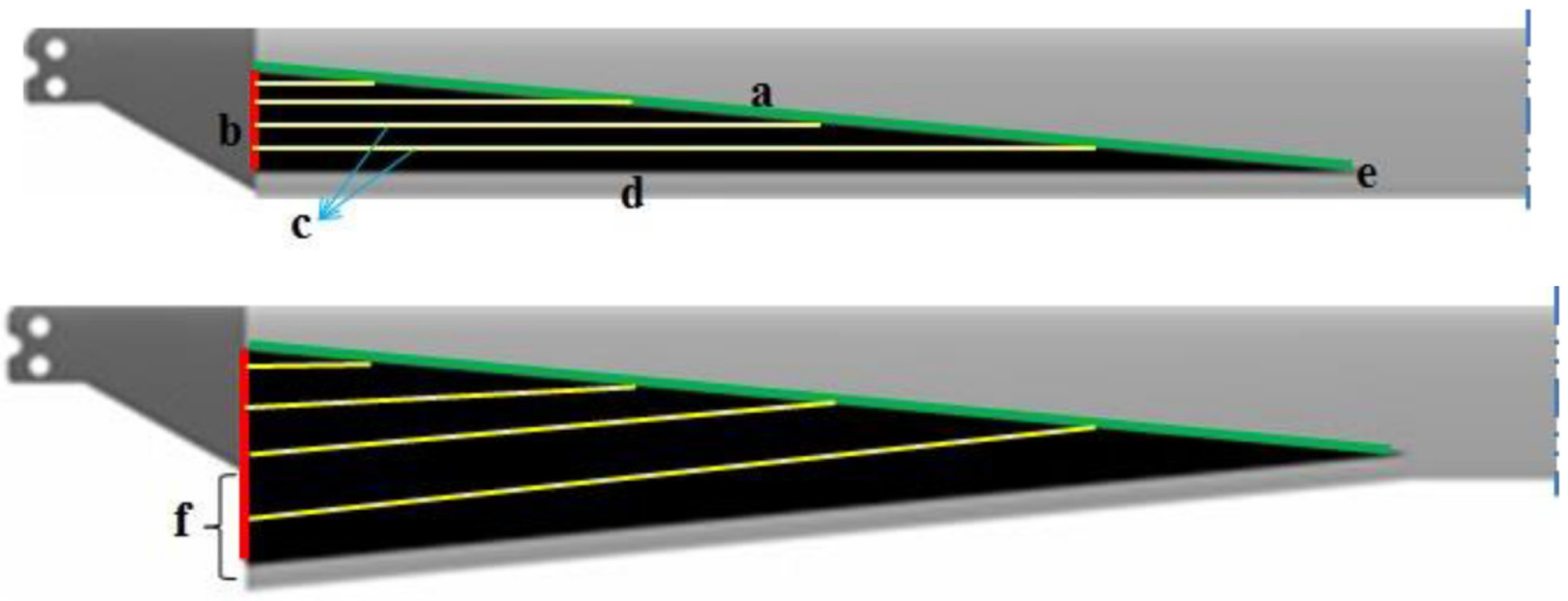
Top view of unmorphed rotor blade (top) and the fully extended rotor blade with a) auxiliary spar (green), b) guidance system (red), c) web stiffeners, d) rear spar, e) hinge, f) maximum chord-extension (Δ _c_).

**Figure 3.**
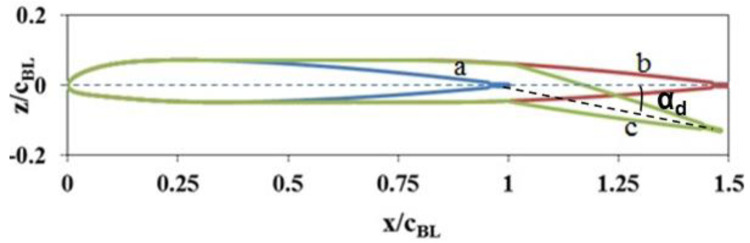
NACA23012tab airfoil with chord-extension and deflection. a) Baseline airfoil, b) with 50% chord-extension, c) with 50% chord-extension and deflection.

It is known that for the so-called “optimum hovering rotor”, the blade chord and the twist distribution of the blade both vary hyperbolically with span length ^15^ as in Figure 1. Based on this principle, in the linearly variable chord-extension morphing concept, the trailing edge is hinged out as shown in Figure 2. In addition to the chord-extension, another feature was also included in the morphing rotor. This involved deflecting the extended chord in a similar manner as would be done with a trailing-edge flap, thereby introducing an effective twist ( Figure 3). Thus, in this concept, the blade chord as well as the blade twist taper down from near the inboard region to a constant value from some point (hinge) in the outboard region.

The structural arrangement for the linearly variable chord-extension morphing is described as follows referring to Figure 2. A rear spar (d) is located in the trailing edge and can be swept back around a hinge (e) which is located at a desired blade radius. At the innermost radial location of the aerodynamic section (at 22% blade radius) this spar is actuated perpendicular to the radial direction by an actuator (b). It is to be noted that the term ‘maximum chord-extension’ refers to the chord-extension at this location.

**Figure 4.**
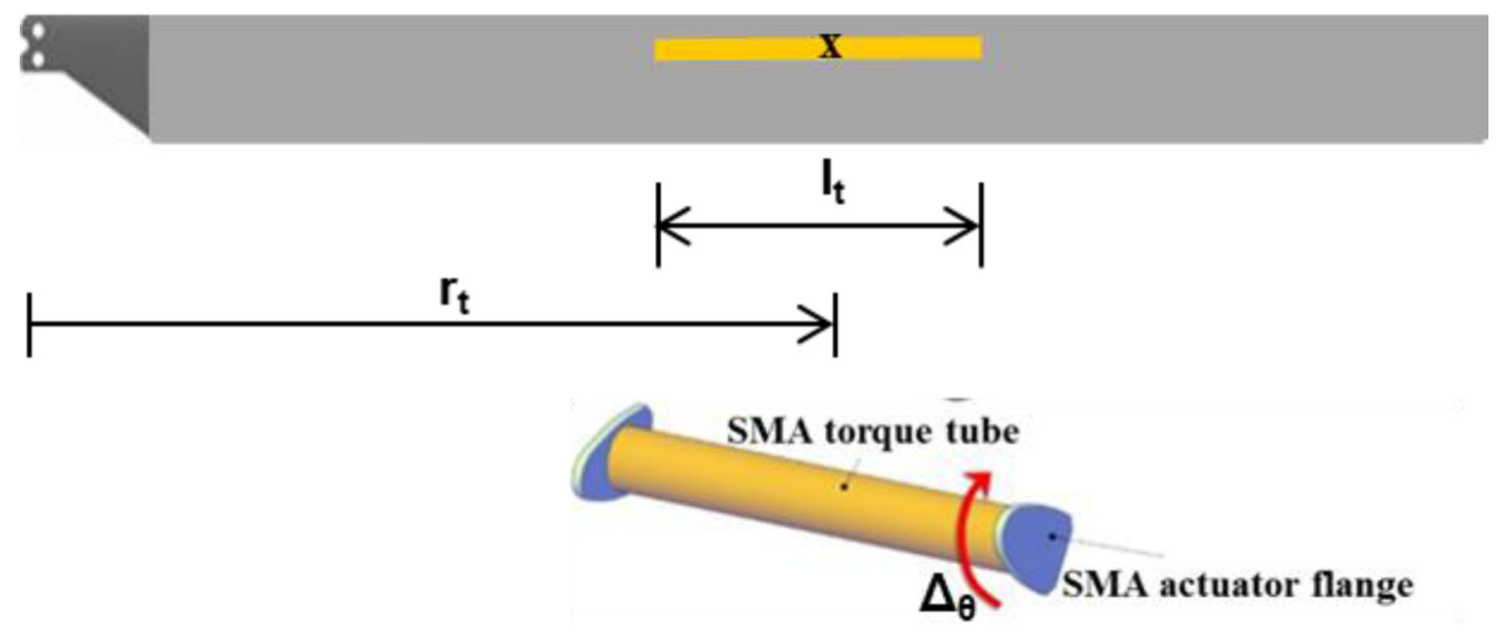
SMA driven twist concept ^3^.

In the torque-tube twist concept, a pre-twisted SMA tube with flanges on either end is inserted into the blade section by removing foam material and connected through the flanges to the leading-edge C-Spar ( Figure 4). By heating the tube to varying degrees when required, the desired twist may be transmitted to the blade.

***Morphing parameters.*** As mentioned earlier, the geometry of the morphed rotor can be described by morphing parameters. The set of morphing parameters for the two morphing concepts in consideration are given in Table 1. It is to be noted that for a particular mission, parameter i1 is fixed for the chord-morphing case while parameters i2 and i3 are expected to be varied during the course of the mission depending on the objectives. Similarly, for the twist morphing case, parameters i1 and i2 are fixed for a particular mission while parameter i3 is expected to be varied over the course of the mission.

**Table 1.** Morphing parameters.

Parameter	Chord-morphing	Twist-morphing
**i1**	Non-dimensional radial location of hinge, r _h_	Non-dimensional radial location of torque-tube center, r _t_
**i2**	Maximum chord extension, Δ _c_	Non-dimensional torque-tube length, l _t_
**i3**	Chord deflection, α _d_	Percent twist of torque-tube, Δ _θ_

### Mission description

**Figure 5.**
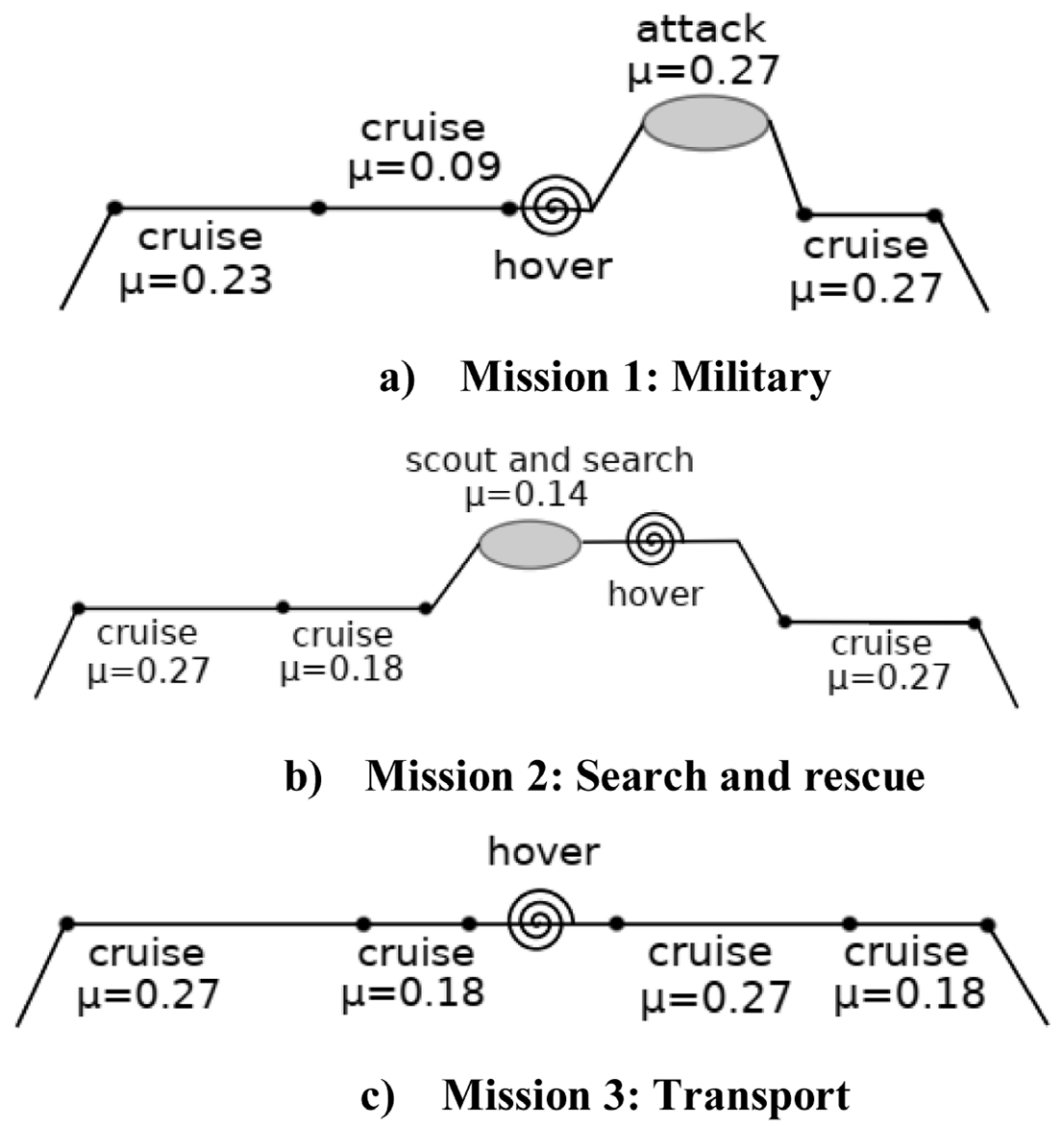
Selected mission profiles.

Helicopters are used for a variety of missions because of their hovering and loitering capacity. Recognizing that the optimum total power requirement is mission dependent, three different mission profiles, namely a military mission, a search and rescue (SAR) mission and a regular transport mission have been selected for this work ^16, 17^. These missions give an overview of typical helicopter applications. The mission profiles are shown in Figure 5. Details of each mission have been taken from said references. Each leg of a specific mission has been given a weightage proportionate to its duration with respect to the overall mission duration. The weightages (w _vi_) are shown in Table 2. Mission 1 has longer hover and low velocity phases compared to the high velocity phase while mission 3 has shorter hover phase compared to the high velocity phase. Mission 2 has a uniformly distributed velocity profile. The altitude variations during the course of the missions are not considered large enough to cause much variations in air density. Hence, for all practical purposes, air density is considered to be constant throughout any mission. The absolute altitudes of flight are not relevant for the relative power analysis of morphing configurations as much as the duration of each leg of the mission.

**Table 2a.** Mission 1 profile.

Velocity (m/s)	Advance ratio μ	Duration (s)	w _vi_ %
0	0.0	1500	25
20	0.09	1796	31
50	0.23	718	13
60	0.27	1800	31

**Table 2b.** Mission 2 profile.

Velocity (m/s)	Advance ratio μ	Duration (s)	w _vi_ %
0	0.0	120	12
30	0.14	250	24
40	0.18	120	12
60	0.27	530	52

**Table 2c.** Mission 3 profile.

Velocity (m/s)	Advance ratio μ	Duration (s)	w _vi_ %
0	0.0	60	7
40	0.18	240	29
60	0.27	530	64

### Optimization problem definition

The linearly variable chord morphing and twist morphing concepts have been individually shown to be effective in improving the overall performance of the rotor ^2, 4^. But this improvement in performance came at the cost of a high elastic twist. The question arises, whether combining the two concepts results in synergizing their benefits, giving rise to better performance with minimum penalty or even reduction of the loads and vibrations. The combined morphing poser, by virtue of the large design space of possible morphing parameter values, lends itself to an optimization study. However, prior to combining the two morphing concepts, a detailed optimization study of the individual concepts for different missions is desired. The optimization algorithm chosen for the current work was the PSO due to its lesser function evaluations compared to other evolutionary algorithms. Details of the PSO implementation used in this work are given in 14, a brief summary of which is given in the sub-section below. The PSO algorithm is combined with S4, the in-house comprehensive analysis code at DLR ^18, 19^, which analyses helicopter rotors in hover and forward flight. S4 provides objective function values to the PSO algorithm.

***Particle swarm optimization.*** In this method, a swarm of particles (population) is, initially, randomly distributed across the design space. The current position of each particle in the swarm ( Equation 1) at any point in time is then updated using a velocity vector ( Equation 2). Throughout the current work, a unit time step is used (Δ *t* = 1).


$$\;x_{i+1}^{p}=x_{i}^{p}+v_{i+1}^{p}\text{Δ}t\;(1)$$

$$v_{i+1}^{p}=wv_{i}^{p}+c_{1}r_{1}\frac{\left(h_{i}^{p}-x_{i}^{p}\right)}{\text{Δ}t}+c_{2}r_{2}\frac{\left(h_{i}^{g}-x_{i}^{g}\right)}{\text{Δ}t}\;(2)$$

**Figure 6.**
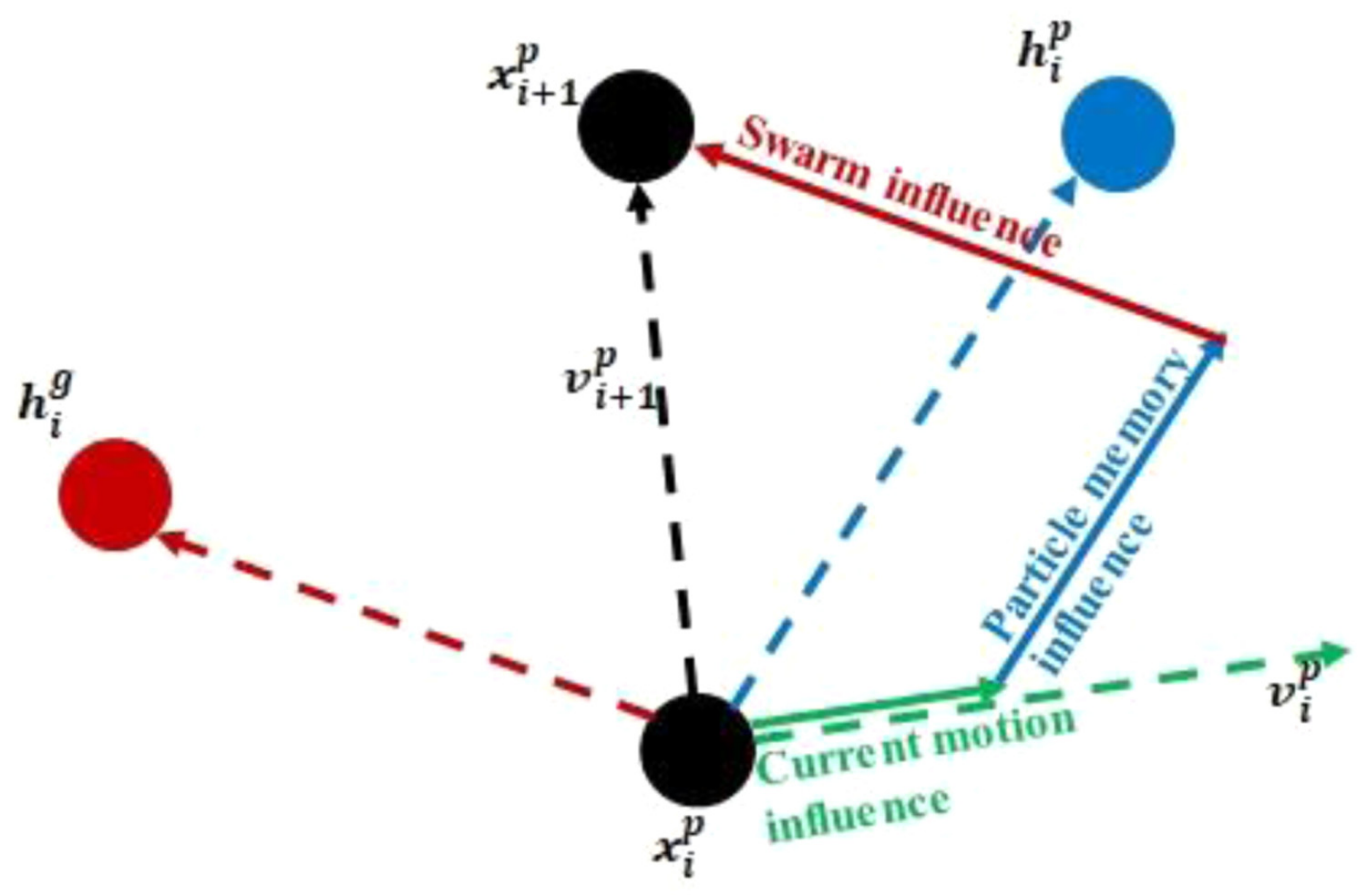
Position and velocity update for a swarm particle.

The above equations are illustrated in Figure 6. Here, $x_{i}^{p}$ and $v_{i}^{p}$ are the position and velocity of particle *p* at iteration *i*; *r*_1_ and *r*_2_ are randomly generated factors ranging from 0 to 1, which differ for each particle and iteration; $h_{i}^{p}$ represents the best ever position of particle *p* where the objective function value is minimum, and $h_{i}^{g}$ corresponds to the global best position up to iteration *i* for the minimal value of the objective function for the whole swarm. *w,* *c*_1_ and *c*_2_ are optimization parameters limited to the 0–1 range. *w* is the inertia weight controlling the exploring abilities of the swarm. Large inertia weights allow for global exploration of the design space. *c*_1_ and *c*_2_ are trust parameters indicating, respectively, how much confidence the current particle has in itself and how much in the swarm.

***Objective function.*** Given the multidisciplinary nature of helicopter rotor analyses, the optimization study is proposed to be multi-objective. The following are the objectives selected for this optimization study: total rotor power required, P (performance criterion), blade tip elastic torsion, Φ (structural criterion) and N/rev hub vertical shear, Fz _4/rev_ (vibration criterion). In mathematical terms,


$${\text{f}}_{1}(\text{X})=\text{P};\;{\text{f}}_{2}(\text{X})=\text{Φ};\;{\text{f}}_{3}(\text{X})={\text{Fz}}_{4/\text{rev}}$$

The rotor power required has been chosen as an objective because the primary goal of the parent project SABRE is to improve the efficiency of the helicopter. The blade tip elastic twist is a critical parameter because it was found to be prohibitively high in the initial morphed designs, because of the change in torsional stiffness. Hence, this parameter was chosen as an objective. The tip elastic twist, being proportional to the root torsional moment, is also indicative of the magnitude of structural loads. The main rotor is the primary contributor of vibratory loads to the fuselage in the form of harmonics of the blade passage frequency. Since the Bo105 rotor, chosen for this work, is 4-bladed, the 4/rev harmonic of its vertical hub load, Fz, is a good measure of its vibratory loads. Thus, three critical aspects of helicopter rotor design, namely, performance, structural loads and vibratory loads, were considered for optimization in this work.

The multi-objective problem can be converted to a single-objective problem by building a composite objective function using the classical weighted sum method ^20^ from the individual normalized, weighted objective functions:


$$\;f=\sum_{i}\frac{f_{i}}{f_{ni}}*w_{i}\;(1)$$

where f is the composite objective function; f _ni_ is the normalizing factor; w _i_ is the weighting factor.

The normalizing factors chosen for the three objective functions are: 350kW (for P), 2.5 deg (for Φ), and 500 N (for Fz _4/rev_).

***Design constraints.*** Constraints are restrictions that must be satisfied to produce an acceptable design. The first requirement set here is that the rotor power required, P, at any velocity for the morphed rotor must be less than the corresponding rotor power (P _BL_) required for the baseline rotor at that velocity. Φ and Fz _4/rev_ have upper constraints too. Thus,


$$\begin{array}{c} 0\;\text{kW}\;<\;\text{P}\;<\;{\text{P}}_{\text{BL}}\\ 0\;\deg\;<\;\Phi \;<\;5\;\deg\\ 0\;\text{N}\;<\;{\text{Fz}}_{4/\text{rev}}\;<\;1000\;\text{N} \end{array}$$

Another requirement is that the rotor must be completely trimmed for any flight condition. This constraint is difficult to be translated into a mathematical form. It is implemented as a Boolean relation checking in an S4 output file for trim confirmation. 

***Design variables.*** The design variables used are the same as the morphing parameters. In the case of chord-morphing, the design variables and their corresponding limits are:


$${\text{X}}_{\text{c}}=\{{\text{r}}_{\text{h}},\;{\text{Δ}}_{\text{c}},\;{\text{α}}_{\text{d}}\}$$

r _h_ ∈ [0.4 - 0.7]; Δ _c_ ∈ [0% - 100%]; α _d_ ∈ [0 ^o^ - 15 ^o^]

The hinge location is limited to a maximum of 0.7 because longer lengths of the rear spar result in higher cross-sectional out-of-plane deformation of the morphed parts of the blade. Hinge locations less than 0.4 do not provide any substantial aerodynamic benefits because of the negligible morphed area. As will be seen in the next section, the dynamic response problem of the rotor is solved in the modal domain. For every change in the rotor structural properties, the modal information has to be recalculated. In order to keep the computational effort to reasonable levels, the design variable domains are discretized as follows:

r _h_ ∈ [0.4 - 0.7] in steps of 0.02
  Δ _c_ ∈ [0% - 100%] in steps of 12.5%
  α _d_ ∈ [0 ^o^ - 15 ^o^] in steps of 5 ^o^

The following are the design variables for the case of twist morphing –


$${\text{X}}_{\text{t}}=\{{\text{r}}_{\text{t}},\;{\text{l}}_{\text{t}}\text{,}\;{\text{Δ}}_{\text{θ}}\}$$

r _t_ ∈ [0.22, 0.39, 0.56, 0.73, 0.9]; l _t_ ∈ [0.0625, 0.125, 0.1875, 0.25, 0.5, 0.75]; Δ _θ_ ∈ [0.00, 0.22, 0.44, 0.66, 0.88, 1]

Thus, the first goal of the current optimization study is to find the combination of morphing parameters of a helicopter rotor which minimizes the performance, structural and vibration objective functions subject to some design constraints, for three different chosen mission profiles.

***Analysis.*** It is to be noted that in the case of chord-morphing, the hinge location is a fixed parameter during a mission while the chord-extension and deflection can be changed as desired. These conditions make the morphed rotor optimization a two-level problem. The outer loop (Lo) decides the hinge location, r _h_, using a mission-level objective function. Within the outer loop, an inner loop (Li) determines the optimum combination of chord-extension, Δc, and chord-deflection, α _d_, for each velocity in a mission using the individual objective function. Thus, the outer and inner loops have their own objective functions and design variables:

Design variables:

**X _cLo_** = {r _h_}; **X _cLi_** = {Δ _c_, α _d_}

Objective function:

Inner loop: f _1_( **X _cLi_**) = P; f _2_( **X _cLi_**) = Φ; f _3_( **X _cLi_**) = Fz _4/rev_

A composite objective function (f _Li_) is built using Equation 1. This composite function is calculated for each velocity in a mission. Since the three objective functions are dissimilar quantities with different orders of magnitude, they are all non-dimensionalized with appropriate normalizing factors so that they can be summed together. The three objective functions are given equal weightage, w _i_ = 0.333

Outer loop: $f_{Lo}=\sum {\left(f_{Li}\right)}_{vi}*w_{vi}$

The outer loop objective function is calculated as the sum of composite objective functions of all velocities in a mission scaled by weighting factors, w _vi_, given in Table 2.

Similarly, the case of twist-morphing is also a two-level problem. However here, two parameters, namely, the radial location and the length of the torque-tube are fixed during a mission while the percent twist of the tube can be changed as desired. So r _t_ and l _t_ are determined in the outer loop while Δ _θ_ for each velocity is calculated in the inner-loop: 


$${\text{X}}_{\text{tLo}}=\{{\text{r}}_{\text{t}},\;{\text{l}}_{\text{t}}\text{\};}\;{\text{X}}_{\text{tLi}}=\{{\text{Δ}}_{\text{θ}}\}$$

The inner and outer loop objectives for the twist-morphing case are determined in the same manner as for the chord-morphing case.

Following trade-off studies for convergence and duration, 10 particles were chosen as the population for the outer loop and 20 particles for the inner loop. The following values were taken for the optimization parameters ( *w,* *c*_1_, *c*_2_): - (0.4, 0.1, 0.1) for the outer loop and (0.5, 0.2, 0.2) for the inner loop. These values satisfy the PSO parameter selection heuristic in 21.

The Bo105 main rotor blade with the airfoil NACA23012tab was selected as the baseline blade. The rotor configuration data is available in open literature ^22^. The rotor had a rectangular blade geometry with a radius of 4.92m and a linear twist of -8deg. The vehicle was powered by two engines with total power of 626kW.

***Rotor comprehensive analysis.*** As already mentioned, the comprehensive analysis tool S4, a high resolution, nonlinear, unsteady aerodynamics code with elastic blade structural model, was used for the analysis of the isolated rotor for hover and forward flight conditions. For structural dynamics of the rotor, first, modal analysis was performed using the finite element method (FEM) based on the Houbolt–Brooks formulation ^23^ which considers flap, lag and torsion degrees for the blade element. Next, for the dynamic response problem, the blade modes were subjected to generalized aerodynamic forces and generalized coupling forces from other modes like flap-torsion and flap-lag. The response problem was then solved using Runge–Kutta fourth order time-integration method until convergence was achieved. S4 had an isolated rotor trim module, where the algorithm adjusted the collective pitch and cyclic pitch control inputs and the rotor shaft angle (for forward flight cases) until a trim goal was met.

Since the dynamic response problem was solved in the modal domain, for every change in the structural properties of the blade, a new modal analysis as required. Variation of the morphing parameters / design variables ( Table 1) results in variation of structural properties of the blade and these are captured in the form of surrogate models. The structural properties of the rotor blade at multiple sections were calculated from 3D finite element models which consider the changes in shape of the section. In order to reduce the costs of the optimization process, the modal information of all the rotor blades derived from different combinations of the morphing parameters were obtained in advance and used when needed by the code. The discretization of the domain space of the design variables for chord-morphing, as seen in the previous section, resulted in 16 x 9 x 4 = 576 surrogate models; while for twist-morphing, discretization resulted in 5 x 6 x 6 = 180 surrogate models.

Since chord-morphing resulted in a change of shape of the baseline NACA23012tab airfoil, each such case had to be treated as a new airfoil. As reported in 2, extensive CFD calculations were made for the two-dimensional aerodynamic coefficients of the modified airfoils, over the range of Mach numbers and angles of attack expected to be experienced by the morphed sections of the rotor. Based on this fresh CFD data, unsteady aerodynamic formulation was synthesized for the aerodynamic coefficients as a function of the morphing parameters. This new formulation was then incorporated into S4. It is to be noted that such an exercise is not required for twist-morphing because it does not result in a change of the airfoil shape.

S4 has been thoroughly validated as can be seen from literature. Data from wind tunnel experiments in the HART II test campaign, which utilized a hingeless Bo105 scaled model rotor, were used to corroborate the blade motion and sectional air loads of S4 ^18, 19^.

The unsteady aerodynamic model for the baseline NACA23012tab airfoil in S4 was derived from data obtained from wind-tunnel tests. The new unsteady aerodynamic model in S4 which was synthesized from data obtained by CFD for the SABRE project was validated for performance and loads ^24^ using data from test campaigns carried out at the NASA Ames Research Center 40-by-80-foot wind tunnel, utilizing the full-scale Bo 105 rotor ^25^. The overall trends of the results from S4 have been found to be in good agreement with the test data.

## Results

For the three missions, two scenarios were considered here – the first one was a simplified single-objective scenario where only the rotor power consumption was taken as the objective function for the inner loop. The second scenario had the composite objective function ( Equation 1) calculated from multiple objectives for the inner loop with equal weightages given for all the objectives.

For comparison purposes, the objective function values for the baseline rotor for a range of velocities from hover to fast forward flight are shown in Table 3. The rotor power, P, followed the familiar bucket curve profile with minimum power required at around 30m/s velocity. The blade tip elastic twist, Φ, gradually increased from hover to high speed. The 4/rev harmonic of vertical hub load, Fz, was negligible at hover and was maximum at low speed transition flight at around 30m/s.

**Table 3.** Baseline rotor.

Velocity, m/s	μ	P, kW	Φ, deg	Fz _4/rev_, N
0	0.0	370	0.46	17
10	0.05	308	0.42	99
20	0.09	238	0.43	594
30	0.14	230	0.53	734
40	0.18	255	0.68	629
50	0.23	310	0.88	473
60	0.27	403	1.15	371

### Linearly variable chord morphing

**Figure 7.**
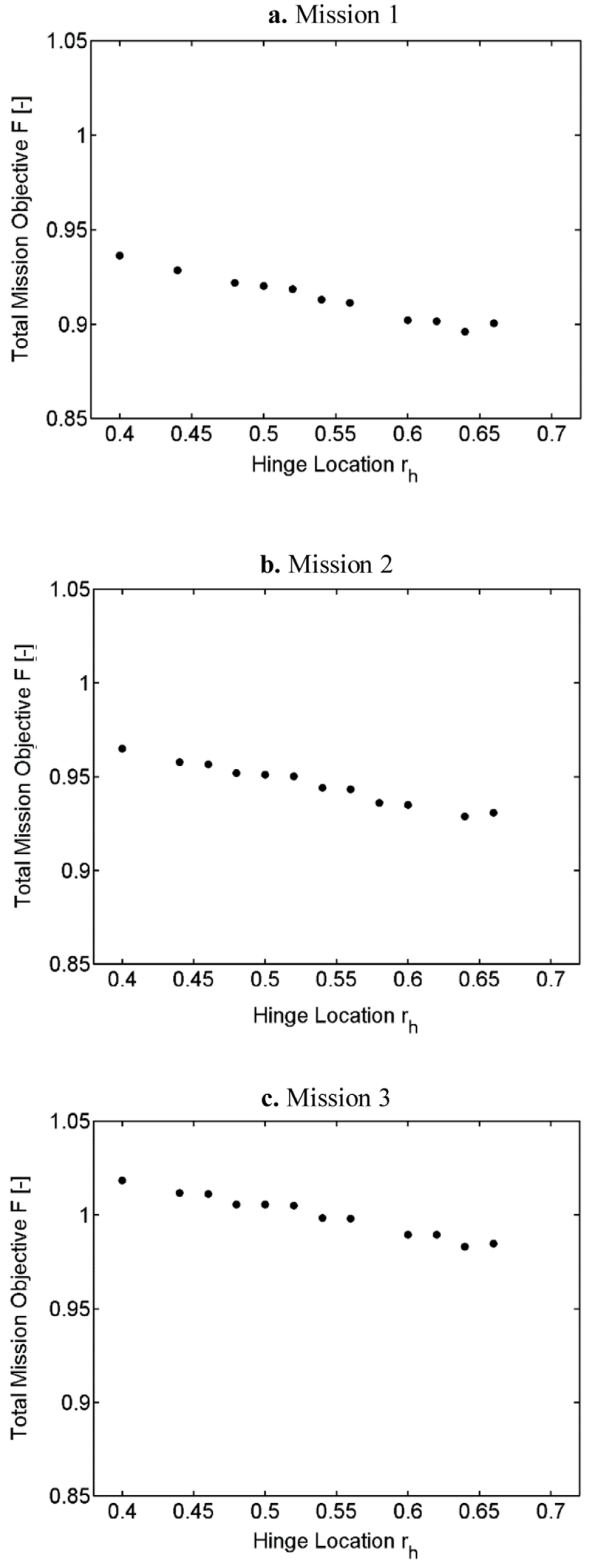
Variation of total mission objective with hinge location in Scenario 1.

***Scenario 1.*** Figures 7a–c show variation of the outer loop objective function (P) with hinge location for the three different missions. To recall, the outer loop objective function calculated the overall mission objective by summing the weighted global objectives at each velocity in the mission.

The three missions showed similar trends. As the hinge location goes outboard, the required rotor power (P) was seen to reduce. This can be explained by the increase in the surface area of the blade which reduces the overall required angle of attack by the rotor blade ^2^. The optimal hinge location was seen to be at around 0.64 for all the three missions. Among the three missions, mission 1 had the least power requirement. This is because of the longer low speed flight duration (bucket region in the power curve). Even though the limit of the hinge location was till 0.7, locations near this outboard region were not seen here because one or more of the design constraint conditions were not met for these values.

**Figure 8.**
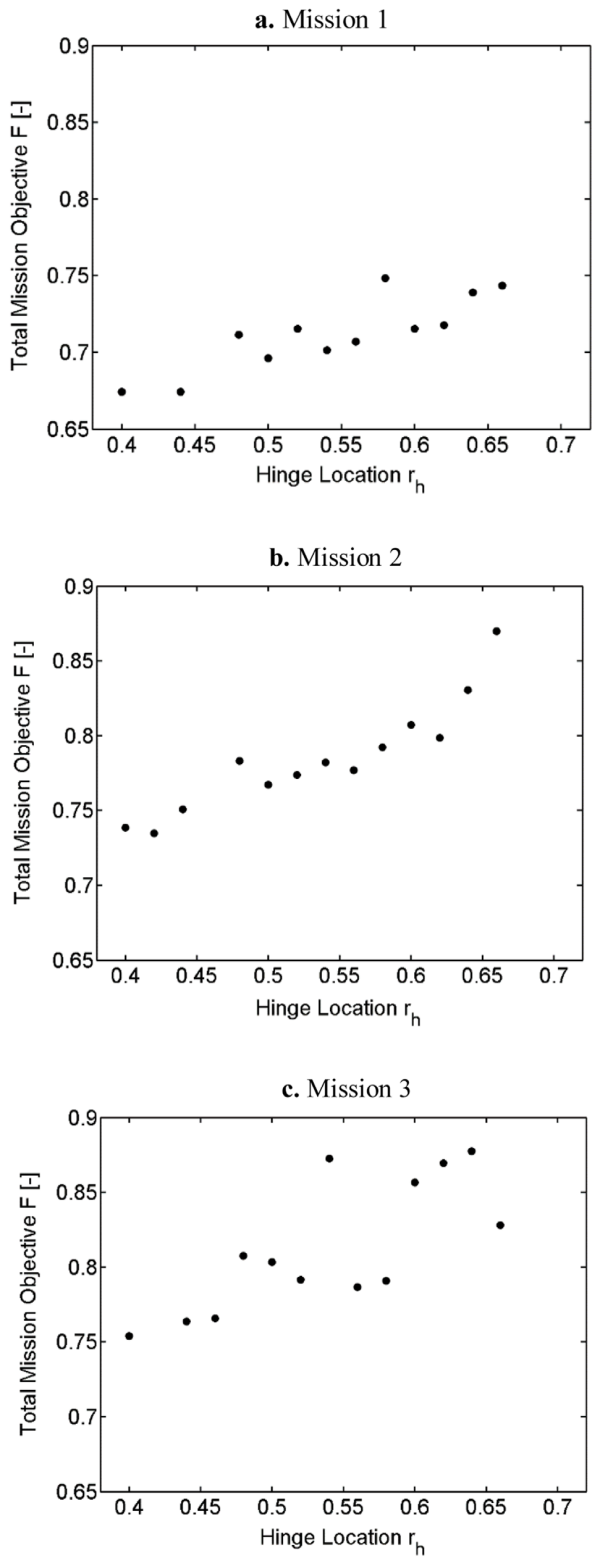
Variation of total mission objective with hinge location in Scenario 2.

***Scenario 2.*** Figures 8a–c also show the variation of the outer loop objective function with hinge location for the three different missions. The objective function used here included the tip elastic twist (Φ) and the hub vertical shear 4/rev harmonic (Fz _4/rev_) apart from the required rotor power (P). All three objectives were given equal weightages.

Even though there were slight deviations, the general trend for all three missions was that the optimum was seen to be moving towards the inboard hinge locations. The optimum hinge locations for all three cases were in the region between 0.4 and 0.45. Mission 1, which had a high percentage of hover and low speed flight, had lower mission objectives because of the lower tip twist and vibratory harmonics at these speeds. The observed deviations could be because of the trim calculations in batch mode which had uniform criteria for all velocities. As a result, some of the cases may not be fully trimmed resulting in slight deviations of the values of the objective functions.

***Other observations.*** Mission 2 was selected for further exploration since it had low and high speeds of similar duration. Table 4a– Table 4b show the variation of the optimal morphing parameters for each velocity leg in mission 2 for scenario 1 and scenario 2, respectively.

**Table 4a.** Mission 2, Scenario 1, morphing parameters.

r _h_	μ	Δ _c,_ %	α _d,_ deg	P, kW	Φ, deg	Fz _4/rev,_ N	P, % savings
0.64	0	100	0	345	4.7	20	6.9
	0.14	100	0	219	4.5	556	4.7
	0.18	6	0	249	1.5	694	2.5
	0.27	0	0	387	2.0	292	3.8

**Table 4b.** Mission 2, Scenario 2, morphing parameters.

r _h_	μ	Δ _c,_ %	α _d,_ deg	P, kW	Φ, deg	Fz _4/rev,_ N	P, % savings
0.42	0	6	0	370	0.7	18	0.0
	0.14	20	0	230	0.8	643	0.0
	0.18	0	0	255	0.8	568	0.0
	0.27	0	0	402	1.4	338	0.1

For scenario 1, a hinge location of 0.64 was found to be the optimal value for the mission. For this hinge location, Table 4a suggests full chord extension for hover and lower velocities for optimum performance and almost nil chord extension for higher velocities. Chord deflections were zero for all velocities. This is because chord deflections resulted in at least one of the design constraints not being satisfied. Comparing with Table 3, it can be seen that the blade tip elastic twist values were much higher than the baseline blade values for this scenario. High blade elastic torsion would require higher pilot collective input angles to offset them and so can be a limiting factor. The vibratory loads were seen to be lower than those for the baseline case. For this mission, compared to the baseline case, the highest power reduction of 6.9% was seen at hover. The overall power reduction for the entire mission was calculated considering the weightages of the individual speeds and was found to be 4.2% for this case.

For scenario 2 ( Table 4b), the optimal hinge location for the mission was at 0.42. The tip elastic twist values were closer to those of the baseline blade values which was a highly positive outcome. Similarly, the vibratory loads were also in the same range as the baseline blade. However, the power required had hardly changed from the baseline values, the reason being an inadequate increase in the morphed area of the blade. This scenario shows that high performance along with reduction in tip elastic twist cannot be achieved merely through hinge-location variance.

To summarize the above findings, for the chord-morphing concept, larger chord-extensions are recommended for hover and low speed flights from a performance perspective. For high-speed flights, lower chord-extensions are recommended. Chord-deflections are not recommended for any flight velocity. A hinge location close to 0.64 can give decent power savings if the blade tip elastic twist can be reduced through careful design by considering factors such as torsional stiffness of the blade in the inboard regions, offset of the shear center from the pitching axis, and geometric twist of the blade.

### Twist morphing

**Figure 9.**
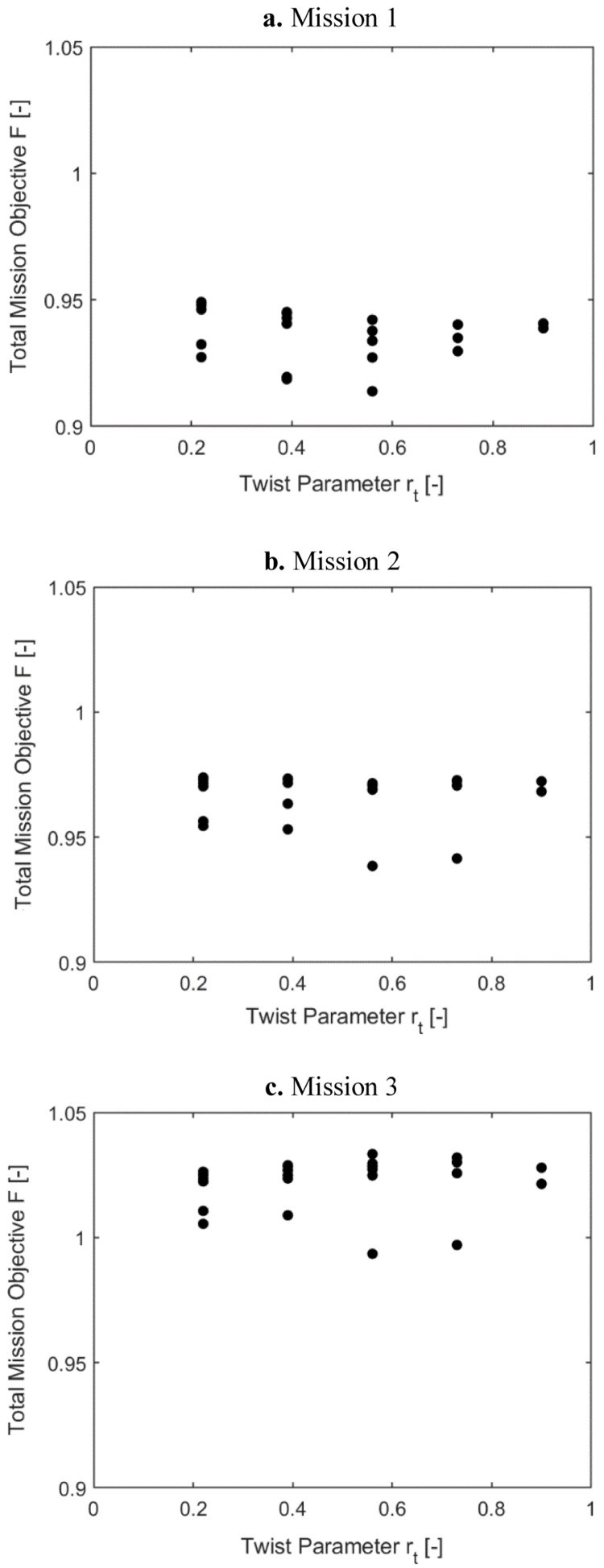
Variation of total mission objective with i1 (r _t_) in Scenario 1.

***Scenario 1.*** For the twist case, there are two outer loop design parameters which need to be fixed for a mission – the radial location of the twist tube (r _t_) and the length of the twist tube (l _t_). Figures 9a–c show the variation of the outer loop mission objective function with respect to the radial location of the twist tube, r _t_ (i1). Each radial location had multiple values of the total mission objective, each corresponding to a different value of the length of the twist tube, l _t_ (i2). Only the minimal values for the radial locations were of interest here, the other data points were shown for completeness. For the three missions, the optimal mission objective occurred at radial locations of the twist tube at 0.56. As the high-speed velocity duration increased in a mission, the mission objective function was also seen to rise.

**Figure 10.**
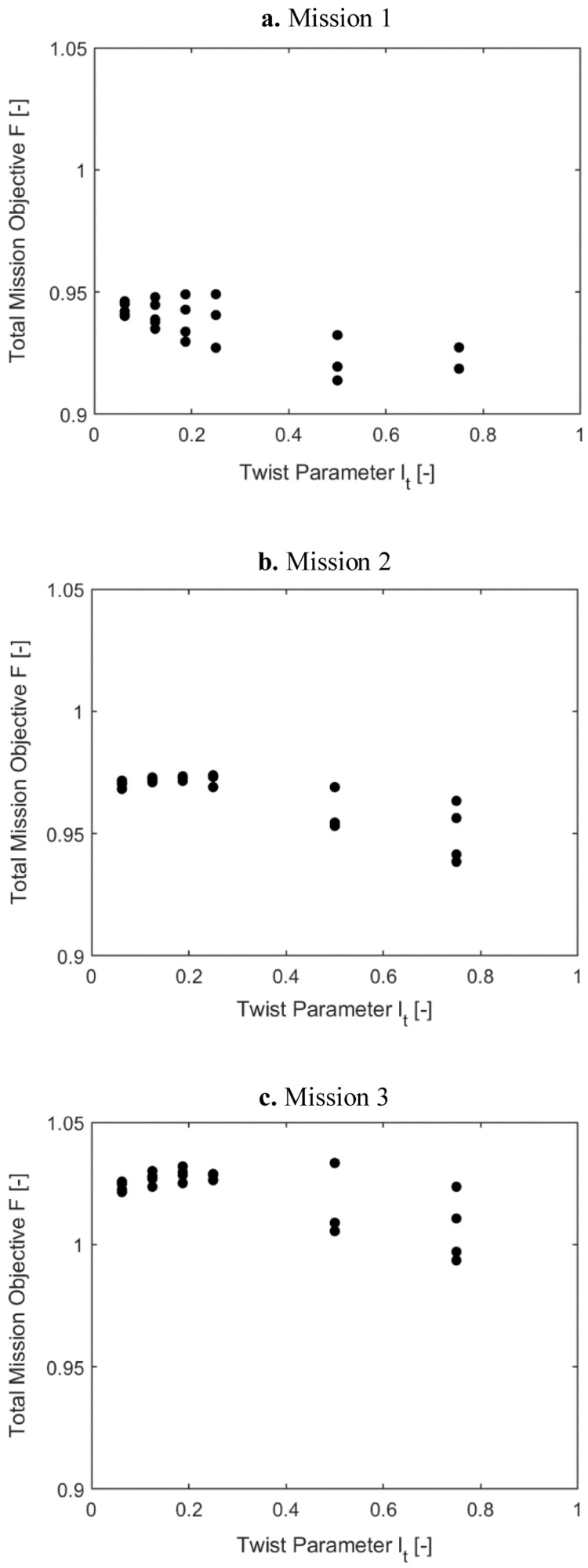
Variation of total mission objective with i2 (l _t_) in Scenario 1.

Figures 10a–c show the variation of the mission objective with respect to the twist tube length for the three missions. The multiple values for each tube length, which have been given for completeness, correspond to a different value of tube radial location. Only the minimal values were of interest here. Tube lengths of 0.5 and 0.75 were seen to give the optimum results.

**Figure 11.**
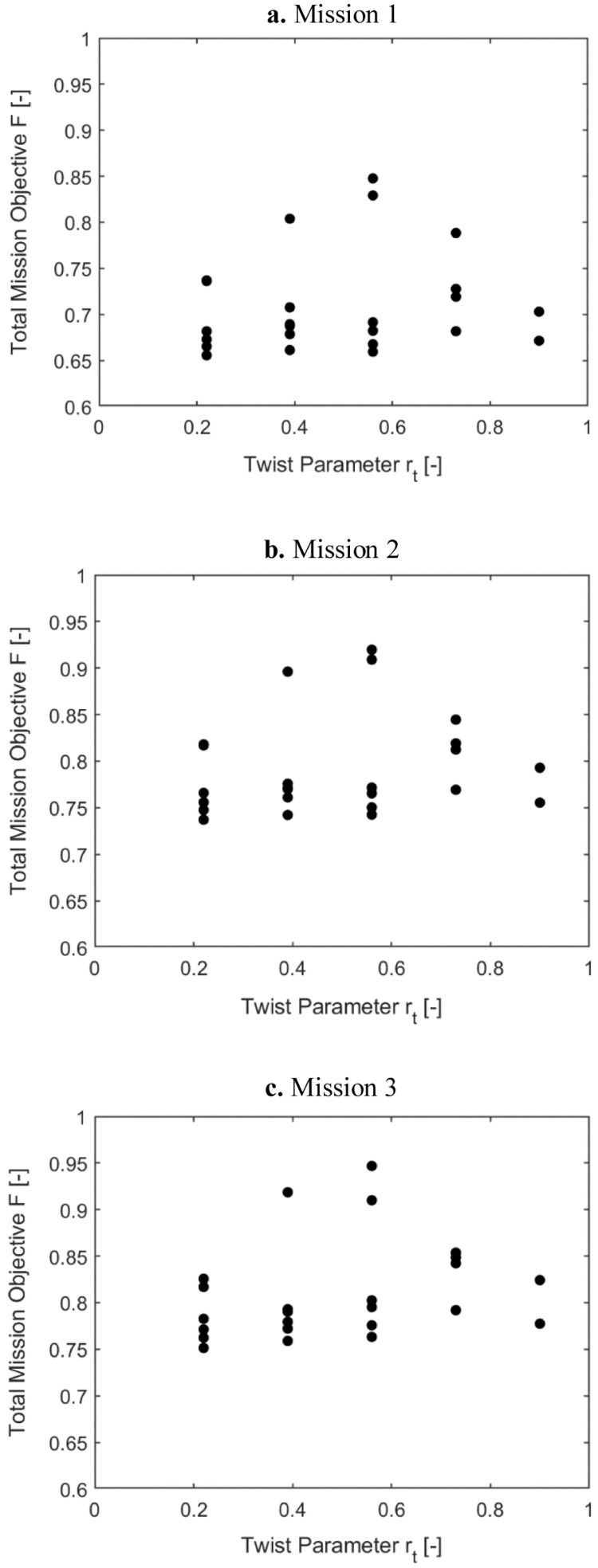
Variation of total mission objective with i1(r _t_) in Scenario 2.

**Figure 12.**
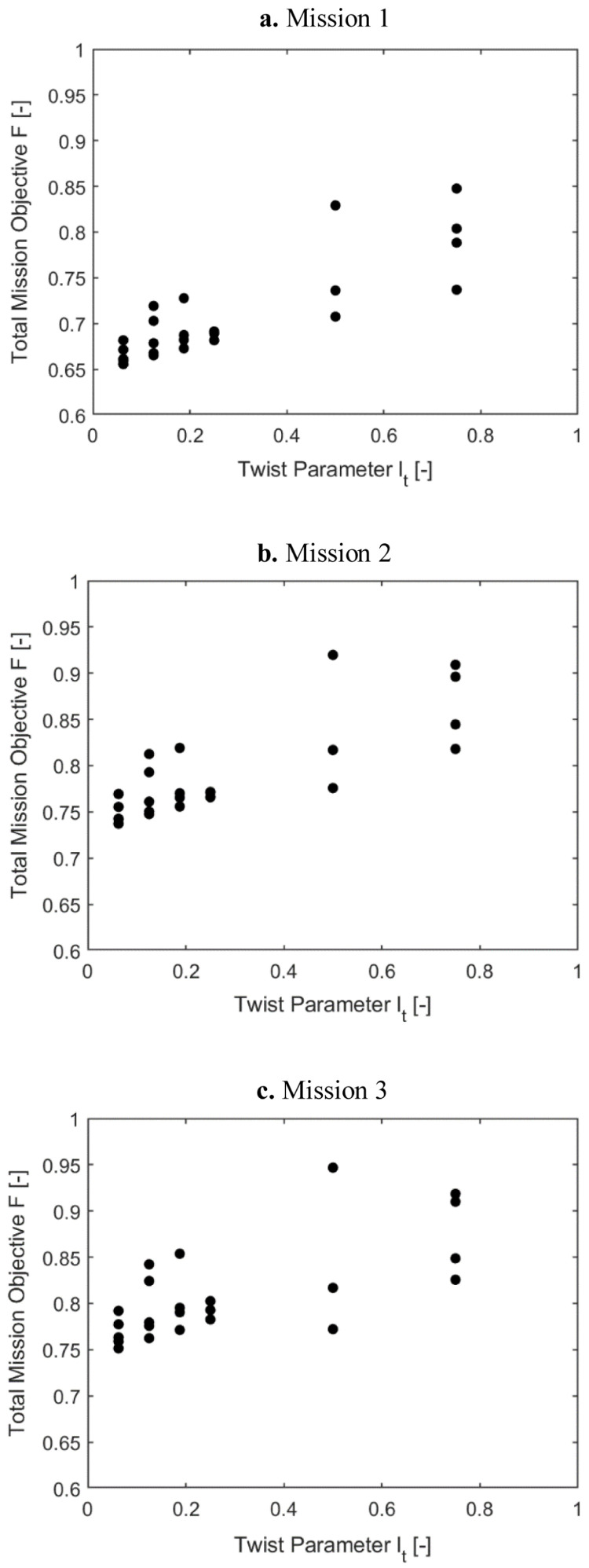
Variation of total mission objective with i2 (l _t_) in Scenario 2.

***Scenario 2.*** Figure 11a–c and Figure 12a–c show the same information as above for scenario 2, where the effect of Φ and Fz _4/rev_ were included with equal weightage as the rotor power. Observing the two sets of plots, it can be concluded that for all the missions, the requirement of optimal elastic twist and vibration shortened the torque-tube lengths to 0.06235 or 0.125 and placed the tube locations more inboard. 

Table 5a, Table 5b shows the variation of optimal morphing parameters for mission 2 for twist morphing. Table 5a gives the values for scenario 1. Optimal radial location of the twist tube was 0.56 and the optimal length of the tube was 0.75. For these fixed values, the delta twist of the tube was seen to decrease as the forward velocity increased. For hover, the full delta was required while for high speeds 22% twist was sufficient. The percentage reduction in rotor power was lower than that for the chord morphing case. For mission 2, at hover, there was a reduction of 11.6% in rotor power compared to the baseline rotor. The overall power reduction for the entire mission was about 3.5%. Vibratory loads were comparable to those of the baseline values. Tip elastic twist, while it was higher than the baseline values, was lower than those for the chord morphing case.

**Table 5a.** Mission 2, Scenario 1, morphing parameters.

μ	Δ _θ_, deg	P, kW	Φ, deg	Fz _4/rev_, N	P, % savings
0	1	327	2.4	39	11.6
0.14	0.44	228	1.7	877	0.9
0.18	0.22	254	1.7	632	0.4
0.27	0.22	392	2.4	408	2.7

**Table 5b.** Mission 2, Scenario 2, morphing parameters.

μ	Δ _θ_, deg	P, kW	Φ, deg	Fz _4/rev_, N	P, % savings
0	0.22	368	0.5	18	0.5
0.14	0.22	230	0.6	740	0.0
0.18	0.22	255	0.7	633	0.0
0.27	0.22	403	1.2	381	0.0

Table 5b gives the optimal morphing parameter values when Φ and Fz _4/rev_ were also considered in the objective function. A radial location of 0.22 was recommended together with a tube length of 0.0625 for this scenario. The delta twist was higher for forward velocities. Even though the tip elastic twist was brought down close to the baseline values, this scenario was of no significant benefit since the power saving compared to the baseline case was negligible.

To summarize the above results, outboard radial locations of 0.56 and 0.73 for the twist tube gave decent reduction in the rotor power albeit with slightly high elastic twist of the blade. Shorter lengths of the twist tube could be used to reduce the tip elastic twist.

### Combined chord and twist morphing

**Figure 13.**
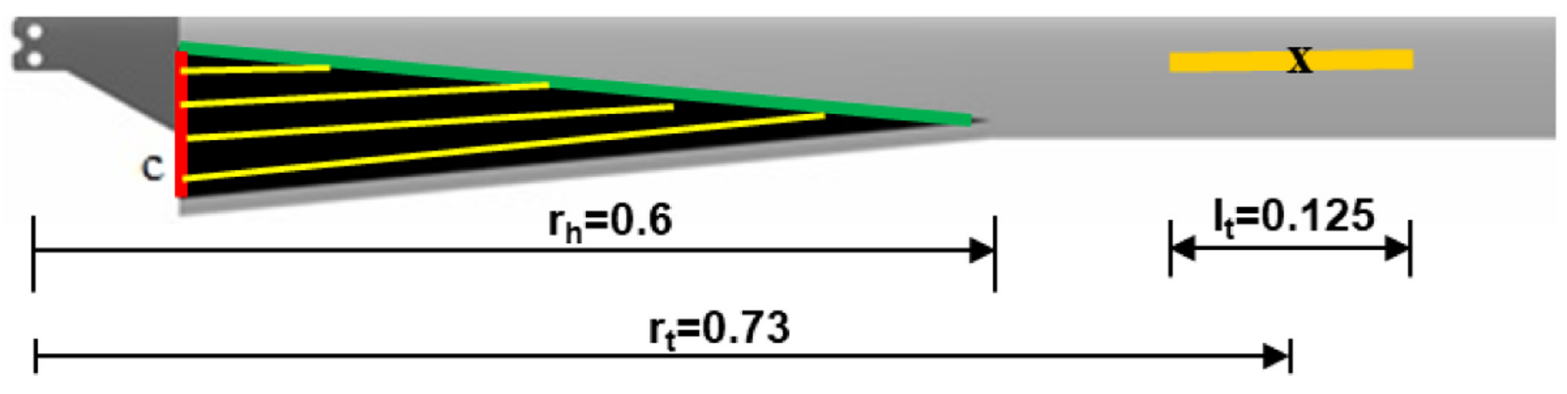
Combined morphing concept.

Based on the trends observed in the previous sections, it can be seen that the optimal configurations of the two morphing concepts could be easily integrated into a single blade with minimal overlapping as shown in Figure 13. From a structural design point of view, it was decided to limit the chord-morphing hinge location to 0.6 in order to reduce the out-of-plane deflection of the morphed airfoil sections. For the twist tube, it was decided to have a length of 0.125 at location 0.73 due to structural constraints. The non-variable morphing parameters for the mission were thus set.

**Figure 14.**
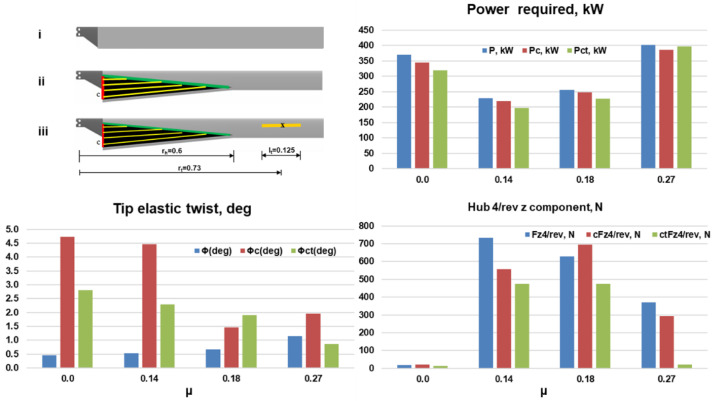
Comparison of rotor analysis results for the baseline, stand-alone chord morphing and the combined morphing blades.

Figure 14 shows a comparison of the power required, tip elastic twist and the vertical hub vibration for the combined morphing blade (subscript ‘ct’) with the baseline blade and the stand-alone chord morphing blade (subscript ‘c’). The rotor with the combined morphing concept compared favorably with the baseline rotor. The power required (green) was less than that for the baseline blade (blue) throughout the mission. The net effective power savings for the complete mission with respect to the baseline blade was 6.8% which was an improvement over the stand-alone chord morphing concept (4.2%). The tip elastic torsion for the combined morphing blade, though higher than that for the baseline blade, was also lower compared to the stand-alone chord morphing case in scenario 1. The hub vibrational loads compared very favorably with the baseline case.

**Figure 15.**
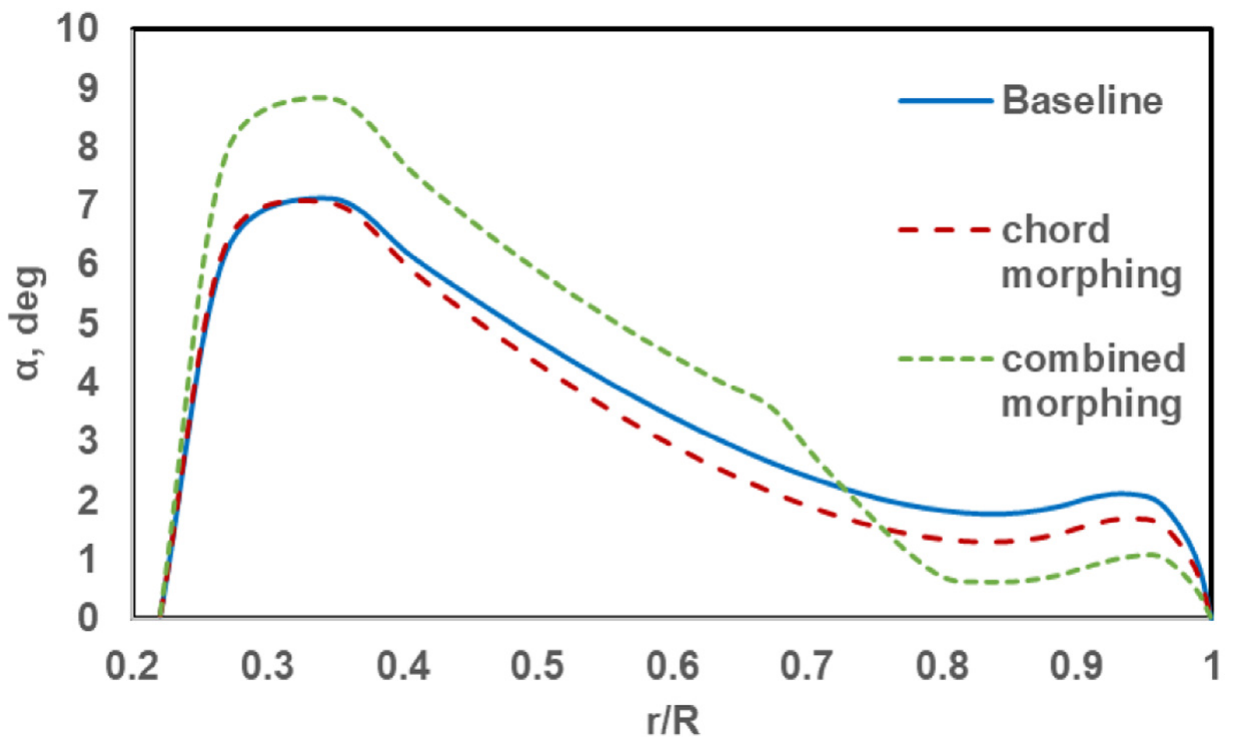
Radial distribution of angle of attack in hover.

The reduction in required power, shown in Figure 14, can be understood by comparing the angle of attack distribution of the three rotors in hover. Figure 15 shows the radial distribution of the angle of attack of the baseline rotor, the chord-morphing rotor and the combined-morphing rotor at the 90° azimuth. Note that for the stand-alone chord-morphing rotor, chord extension was 100% while for the combined-morphing rotor, chord extension was 50%. For the chord-morphing case, the sections from r = 0.35R till r = 0.95R had less angles of attack than those for the baseline case. On average, the reduction was about 0.5°. This reduced the profile drag of a rather large part of the rotor blade and hence, reduced the overall required power. In the combined-morphing rotor, the variation of the angle of attack across the blade was redistributed with a sharp reduction in the final 25% of the blade after the torque-tube. On average, the reduction from the baseline blade was about 1.2° in this region. To compensate for the reduced lift in this region, the angles of attack in the inboard 50% of the blade (from r = 0.28R to r = 0.70R) show an average increase of 1.2°, thus increasing the lift in this region. The profile drag of a rotor blade had its highest contribution from the outboard region where the dynamic pressure was the highest. For the combined-morphing blade, profile drag in the outboard region was drastically reduced because of the low angles of attack, thus reducing the overall power required.

It was observed in the Campbell diagrams of the lower chord-extension blades that there was a strong flap-torsion coupling at rotor design speed. This resulted in lower chord-extension rotors not trimming at high speeds. With further design changes to prevent the flap-torsion coupling at rotor design speeds, it should be possible to have higher power savings with the combined morphing concept.

## Conclusions

Linearly variable chord morphing and torque-tube twist morphing for helicopter rotor blades were studied for their mission-based optimal geometric parameters. For this, an evolutionary algorithm, PSO, was combined with a high- resolution helicopter rotor comprehensive analysis code, S4. A fully elastic rotor blade was modelled in an unsteady aerodynamic environment. It was found that the two concepts could have non-overlapping optimal conditions. By combining both concepts into a single rotor blade, better performance than the baseline blade was achieved, while the penalty in elastic torsion and hub vibrational load were within reasonable limits. The combined concept also gives better performance than the individual stand-alone concepts. A couple of factors which limit the performance of the combined concept blade are to be noted: one is the flap-torsion coupling at the design rotor speed for the lower chord-extension cases. The other factor is the low torsional stiffness of the chord-morphing region of the blade, which resulted in high elastic twist in the blade. Improving these two design aspects can result in smoother trends in the optimization results, and can lead to further improvements in the rotor efficiency.

## Abbreviations

BL                        Baseline

Fz _4/rev_                  4/rev harmonic of vertical hub load, Fz

l _t_                           torque-tube length, non-dimensional wrt R

P                           Main rotor required power, kW

r                            Rotor blade radius variable, m

r _h_                          Non-dimensional radial location of hinge

r _t_                           Radial location of torque-tube center

R                           Radius of rotor, m

α                           Angle of attack, deg

α _d_                          Chord deflection, deg

Δ _c_                          Maximum chord extension as a percentage of baseline chord

Δ _θ_                          Percent twist of torque-tube

μ                            Advance ratio

Φ                           Blade tip elastic twist, deg

## Data availability

### Underlying data

The program and part of the data (pertaining to structural properties of the chord-morphed sections) used in this work are owned by the lead author’s employer, the German Aerospace Center (DLR), and are restricted by the organization to internal use only. Other data (structural properties of the baseline blade and those of the twist-morphed sections) are held under restriction by third parties and are not available for publication. All data underlying the results and necessary for review are available as part of the article or in relevant references.
