# Supplementary material for: Mission-based optimal morphing parameters for rotors with combined chord and twist morphing
Source: Open Res Eur. 2022 Jun 7;1:121. Originally published 2021 Oct 12. [Version 2] doi: 10.12688/openreseurope.14060.2 (PMC10445829; doi:10.12688/openreseurope.14060.2)
Supplement: Peer review history

# Peer review: Reviewer response for version 2

**DOI:** 10.21956/openreseurope.16049.r29481

**Published:** 2022-06-14

**Authors:** Wen Zhong Shen

**Affiliations:** 1 College of Electrical, Energy and Power Engineering, Yangzhou University, Yangzhou, China

The authors answered all my questions with satisfaction and I have no further comments.

Is the study design appropriate and does the work have academic merit?

Yes

Is the work clearly and accurately presented and does it cite the current literature?

Yes

If applicable, is the statistical analysis and its interpretation appropriate?

Not applicable

Are all the source data underlying the results available to ensure full reproducibility?

Partly

Are the conclusions drawn adequately supported by the results?

Yes

Are sufficient details of methods and analysis provided to allow replication by others?

Partly

Reviewer Expertise:

NA

I confirm that I have read this submission and believe that I have an appropriate level of expertise to confirm that it is of an acceptable scientific standard.

# Peer review: Reviewer response for version 2

**DOI:** 10.21956/openreseurope.16049.r29482

**Published:** 2022-06-14

**Authors:** Manfred Hajek

**Affiliations:** 1 Institute of Helicopter Technology, Technical University of Munich, Munich, Germany

I have read the new version and checked the authors’ response to my comments. Sufficient improvement has been made so that I can agree to indexing without further request for modification.

Is the study design appropriate and does the work have academic merit?

Partly

Is the work clearly and accurately presented and does it cite the current literature?

Partly

If applicable, is the statistical analysis and its interpretation appropriate?

Not applicable

Are all the source data underlying the results available to ensure full reproducibility?

No source data required

Are the conclusions drawn adequately supported by the results?

No

Are sufficient details of methods and analysis provided to allow replication by others?

No

Reviewer Expertise:

NA

I confirm that I have read this submission and believe that I have an appropriate level of expertise to confirm that it is of an acceptable scientific standard.

# Peer review: Reviewer response for version 1

**DOI:** 10.21956/openreseurope.15151.r27792

**Published:** 2021-10-27

**Authors:** Manfred Hajek

**Affiliations:** 1 Institute of Helicopter Technology, Technical University of Munich, Munich, Germany

**General remarks:**

 The paper lacks a discussion of the state of the art in order to clearly identify where the authors’ contribution is complementary to the publications in the literature list. This is an essential element of good practice in scientific writing.

**Detailed comments per page:**

 Page 1:

- First line: add “of” between “geometry” and  “rotor blades …”
- “Results”: How was the performance variation quantified? Provide a figure for the performance gain per mission and/or per speed of flight.

Page 3:

- It is not clear enough, which flight regime was regarded (hover, forward flight, low/high speed, or both).
- Are the morphing concepts active or passive?
- Which frequencies or rotor harmonics were applied?
- Before the section named “Methods”, a discussion of the state of the art is missing.

Page 4:

- After “… outboard region.”: it is unclear where the flap is implemented.
- Para beginning with “In the torque-tube …”: Which range of twist angles was applied? Was it active or passive and which frequency was applied?
- Variables should be written in italics to identify them in the text. This holds for the whole text.

Page 6, Section “Optimization …”:

- The statement “… came at the cost of a high elastic twist” lets unclear which type of morphing is meant, variable chord or twist morphing or both.

Page 7, first line:

- Why is only the F_z component considered in the vibration criterion? F_x and F_y are not guaranteed to be small. There should at least be a statement in the results section that gives a qualitative assessment of their effect.

Page 8:

- “… data is available in literature (22)”: This reference is not public, better cite the SABRE deliverable D1.1 which is public and contains all relevant data.
- Section “Rotor comprehensive analysis”, line 2: what means “high resolution”? Quantify!
- The structural properties of the baseline blade are not provided. A description of changes to these structural changes is missing. How is the structural model composed? Which beam theory was applied and which spatial discretization was used? How was the structural model validated?
- Surrogate models: Description is missing.
- Data for the CFD model are missing/not referenced.
- The inflow model description is missing.

Page 9:

- The determination of the optimal hinge location is questionable: The data points in Fig. 7 do not allow to identify the minimum as precisely as mentioned in the text.
- The angle of attack for the data points in Fig. 7 are not provided.
- A definition of the Total Mission Objective is missing. How is it defined?
- Fig.7 should combine all results in one chart.

Page 10:

- Top of page: “… may not be fully trimmed …”: The untrimmed cases should not be used in Fig. 8. Otherwise the data points cannot be compared.
- Like in Fig. 7, the determination of the minimal hinge location is questionable.

Page 11:

- Table 4a: Explain why values for P% decrease while µ is increasing.
- Table 4b:  Explain why values for P% remain  constant while µ is increasing.
- Paragraph beginning “Figures 10a-c …”: The statements in this para are not supported by fig. 10a-c.
- Paragraph beginning “To summarize …”: Statement on elastic twist is insufficient, it should better be displayed in a figure.

Page 12 and 13:

- Plots are not readable without legend.

Page 14,  first Para:

- F_z_4/rev at µ = 0.27 in Fig. 14 is conspicuously low especially when considering that quasi-static morphing mechanisms are involved (and not active blade morphing). Consequently, a description of underlying factors is deemed essential here and should be provided by the authors (much like the bevior of P is explained in the following para.
- Table 5a: What is Δ _Θ_ = 1 corresponding to?
- Tables 5a and 5b: Why is µ unequally spaced?

Page 15, Fig. 15:

- Add details (e.g. µ, flight case) to the caption.

Page 16:

- Abbreviations table covers only a part of the names of entities used in this text.

Is the study design appropriate and does the work have academic merit?

Partly

Is the work clearly and accurately presented and does it cite the current literature?

Partly

If applicable, is the statistical analysis and its interpretation appropriate?

Not applicable

Are all the source data underlying the results available to ensure full reproducibility?

No source data required

Are the conclusions drawn adequately supported by the results?

No

Are sufficient details of methods and analysis provided to allow replication by others?

No

Reviewer Expertise:

Rotorcraft aeromechanics, dynamics of rotating blades, aerodynamics of rotors

I confirm that I have read this submission and believe that I have an appropriate level of expertise to state that I do not consider it to be of an acceptable scientific standard, for reasons outlined above.

# Response

**Published:** 2022-05-25

**Authors:** Rohin Majeti

**Affiliations:** German Aerospace Center (DLR), Germany

Author responses in italics throughout.

**General remarks:**

 The paper lacks a discussion of the state of the art in order to clearly identify where the authors’ contribution is complementary to the publications in the literature list. This is an essential element of good practice in scientific writing.  - *Updated state of the art. Done*

**Detailed comments per page:**

 Page 1:

- First line: add “of” between “geometry” and  “rotor blades …”

Response: *Done*

- “Results”: How was the performance variation quantified? Provide a figure for the performance gain per mission and/or per speed of flight.

Response: *Done*

 Page 3:

- It is not clear enough, which flight regime was regarded (hover, forward flight, low/high speed, or both) – *a mission includes all the regimes.*
- Are the morphing concepts active or passive? - *passive*
- Which frequencies or rotor harmonics were applied? – *passive morphing*
- Before the section named “Methods”, a discussion of the state of the art is missing. - *Done*

Response: *The introduction has been updated to address each of the above comments. As has been explained later in the article, the performance over a whole mission which includes a mix of flight regimes has been considered. The morphing concept is a passive, quasi-static one. The state-of-the-art discussion has been updated.*

 Page 4:

- After “… outboard region.”: it is unclear where the flap is implemented. – *flap hinge is a variable determined by solving the optimization problem.*
- Para beginning with “In the torque-tube …”: Which range of twist angles was applied? Was it active or passive and which frequency was applied? – *passive morphing like in the case of chord morphing.*
- Variables should be written in italics to identify them in the text. This holds for the whole text. – *Done*

Response: *All the above comments have been addressed within the text. The flap hinge is a variable determined by solving the optimization problem. Torque-tube morphing is also a passive case like in the case of chord morphing; here the maximum twist angle is -16deg.*

 Page 6, Section “Optimization …”:

- The statement “… came at the cost of a high elastic twist” lets unclear which type of morphing is meant, variable chord or twist morphing or both.

Response: *The statement is relevant for both the morphing concepts.*

 Page 7, first line:

- Why is only the F_z component considered in the vibration criterion? F_x and F_y are not guaranteed to be small. There should at least be a statement in the results section that gives a qualitative assessment of their effects.

Response: *It is true that Fx and Fy components of the hub loads can also be significant. The Fz component contributes the most to the vibration in the fuselage and has been considered here to begin with. In follow up studies, the other components are also being considered.*

 Page 8:

- “… data is available in literature (22)”: This reference is not public, better cite the SABRE deliverable D1.1 which is public and contains all relevant data.- *Done*
- Section “Rotor comprehensive analysis”, line 2: what means “high resolution”? Quantify! - *Done*
- The structural properties of the baseline blade are not provided. A description of changes to these structural changes is missing. How is the structural model composed? Which beam theory was applied and which spatial discretization was used? How was the structural model validated? – *Done*
- Surrogate models: Description is missing. - *Done*
- Data for the CFD model are missing/not referenced. - *Done*
- The inflow model description is missing. - *Done*

Response: *The text has been updated taking the above comments into consideration. It may be noted that the focus of the current paper is on the optimization problem and its solution methodology.*

 Page 9:

- The determination of the optimal hinge location is questionable: The data points in Fig. 7 do not allow to identify the minimum as precisely as mentioned in the text.
- The angle of attack for the data points in Fig. 7 are not provided.
- A definition of the Total Mission Objective is missing. How is it defined? - *Done*
- Fig.7 should combine all results in one chart. – *Done*

Response: *Fig. 7 has been updated to include all results in one chart and the trimmed points were extended up to hinge locations of 0.68R by increasing the azimuthal resolution and minor adjustments in structural properties. The Total Mission Objective is defined in Eq. 5. The total mission objective has a decreasing trend towards outboard location of the morphing hinge until 0.64R after which it slightly increases. The angle of attack point was given with reference to Ref. 2 (section 3.4) where the example of hover condition was used to explain the possible cause of power reduction.*

 Page 10:

- Top of page: “… may not be fully trimmed …”: The untrimmed cases should not be used in Fig. 8. Otherwise the data points cannot be compared. - *Done*
- Like in Fig. 7, the determination of the minimal hinge location is questionable. - *Done*

Response: *Using a combination of increased azimuthal resolution and minor adjustments in structural properties, a few of the earlier not-so-trimmed cases were improved and a few points up to hinge location of 0.68R added. The total mission objective has a decreasing trend towards outboard locations of the morphing hinge. This result serves to conclude that if the elastic twist of the blade and hub vibration levels are to be kept lower than or comparable to those of the baseline blade simultaneously, the extendable chord region has to be kept minimum which in turn reduces the potential for power reduction. *  

 Page 11:

- Table 4a: Explain why values for P% decrease while µ is increasing. - *Done*
- Table 4b:  Explain why values for P% remain constant while µ is increasing. – *already given in the text*
- Paragraph beginning “Figures 10a-c …”: The statements in this para are not supported by fig. 10a-c.
- Paragraph beginning “To summarize …”: Statement on elastic twist is insufficient, it should better be displayed in a figure.

Response: *Text and figures have been updated taking above comments into consideration*  

 Page 12 and 13:

- Plots are not readable without legend. - *Done*

Response: *Plots have been updated to highlight the important data*   Page 14,  first Para:

- F_z_4/rev at µ = 0.27 in Fig. 14 is conspicuously low especially when considering that quasi-static morphing mechanisms are involved (and not active blade morphing). Consequently, a description of underlying factors is deemed essential here and should be provided by the authors (much like the bevior of P is explained in the following para.
- Table 5a: What is Δ _Θ_ = 1 corresponding to? - - *16deg*
- Tables 5a and 5b: Why is µ unequally spaced? – *Pre-defined search and rescue mission taken from refs 24, 25.*

Response: *Text has been updated considering the above comments*  

 Page 15, Fig. 15:

- Add details (e.g. µ, flight case) to the caption.

Response: *As indicated in the caption, the flight case is that of hover (µ = 0.0).* Page 16:

- Abbreviations table covers only a part of the names of entities used in this text.

Response: *Done*

# Peer review: Reviewer response for version 1

**DOI:** 10.21956/openreseurope.15151.r27794

**Published:** 2021-10-18

**Authors:** Wen Zhong Shen

**Affiliations:** 1 College of Electrical, Energy and Power Engineering, Yangzhou University, Yangzhou, China

The paper presents a design methodology for helicopter rotors with morphing blade. A chord morphing, a twist morphing, and a combined chord and twist morphing technique were used for the optimizations. Analyses on the designed results were made with respect to power, blade tip elastic torsion and hub vertical shear. It was found that the combined chord and twist morphing technique was spurious over the single chord or twist morphing technique. The paper is clearly written with sufficient detail and interest to read. The study was well designed and can be considered as a good example for designing other rotors than helicopter rotors. This reviewer has a number of questions or comments that can be considered to improve the paper.

1. Abstract: The authors mentioned “change the shape of blades in different regimes” in Background, while it is only “different missions” used in Methods. It needs some clarification whether the different regimes in the 3 missions were considered separately in the design.
2. Table 1, i2: The maximum chord extension seems a dimensional variable. If it is nondimensional, please indicate the reference chord. Same comment for the other non-dimensional parameters.
3. Equation 1: What is i? Mission or regime? Please specify.
4. Design constraints: “the rotor must be completely trimmed”. It would be good to indicate what parts of rotor were trimmed.
5. Page 7, Analysis: The notations in the inner and outer loops are confusing.
6. Page 8: “10 particles” and “20 particles” don’t seem a lot. Any justification?
7. Page 8: Can the chord length of the B105 rotor be provided?
8. Page 8, Left column, the last 3rd line: “as” to “is”.
9. Page 8: CFD needs to be given with more detail. Is it an unsteady code?
10. Page 8 “Based on this fresh CFD data, unsteady aerodynamic formulation”: How are the dynamic effects (such as dynamic stall) considered?
11. Some references are difficult to be accessed.
12. Results: The objective function was indicated for the inner loop. No information is given for the outer loop.
13. Figure 7: Why is the hinge location same for a complete mission as there are different regimes in a mission?
14. Page 11, Combined chord and twist morphing: Some information about the integration is useful to be given as there are inner and outer loops in both techniques.
15. Abbreviations: Some symbols are not listed. Please complete the list.

Is the study design appropriate and does the work have academic merit?

Yes

Is the work clearly and accurately presented and does it cite the current literature?

Yes

If applicable, is the statistical analysis and its interpretation appropriate?

Not applicable

Are all the source data underlying the results available to ensure full reproducibility?

Partly

Are the conclusions drawn adequately supported by the results?

Yes

Are sufficient details of methods and analysis provided to allow replication by others?

Partly

Reviewer Expertise:

NA

I confirm that I have read this submission and believe that I have an appropriate level of expertise to confirm that it is of an acceptable scientific standard, however I have significant reservations, as outlined above.

# Response

**Published:** 2022-05-25

**Authors:** Rohin Majeti

**Affiliations:** German Aerospace Center (DLR), Germany

Author responses in italics throughout.

 Response to Reviewer 1 comments:

- Abstract: The authors mentioned “change the shape of blades in different regimes” in Background, while it is only “different missions” used in Methods. It needs some clarification whether the different regimes in the 3 missions were considered separately in the design.

Response: *Yes, as can be seen from the results later on, blade design (or blade shape) varies with flight regime in a mission. Within a flight regime, the blade shape remains constant.* *For example, in a mission, when the vehicle changes from one flight regime to another, say from hover to low-speed flight of 20m/s, the shape of the blade might have to change, say from 100% chord-extension to 50% chord-extension. During the entirety of hover, it retains the 100% chord-extension and during the entire 20m/s flight, it retains 50% chord-extension. The change in shape happens during the transition.* *The statement has been modified from “change the shape of blades in different regimes” to “change the shape of blades **between** different regimes” in a quasi-static manner.*  

- Table 1, i2: The maximum chord extension seems a dimensional variable. If it is nondimensional, please indicate the reference chord. Same comment for the other non-dimensional parameters.

Response: *Table 1, i2 is a non-dimensional parameter given as a percentage of blade chord. The description has been updated in the table. The reference chord has been added to the blade parameters under the section ‘Analysis’..*

- Equation 1: What is i? Mission or regime? Please specify.

Response: *Equation 1 is the summation of the individual objectives f _1_, f _2_ and f _3_. ‘i’ is the index for 1 to 3.*

- Design constraints: “the rotor must be completely trimmed”. It would be good to indicate what parts of rotor were trimmed.

Response: *Trim, here, refers to the set of control inputs to keep the rotor in equilibrium in a particular flight condition.*

- Page 7, Analysis: The notations in the inner and outer loops are confusing.

Response: *Text has been updated to make the notations clearer.*

- Page 8: “10 particles” and “20 particles” don’t seem a lot. Any justification?

Response: *Optimal swarm size is problem-dependent. A smooth search space will need fewer particles than a rough surface to locate optimal solutions. Swarm sizes of n _s_* *∈*  *[10, 30] have been shown to give optimal solutions for single objective problems. For the current problem, 10 and 20 particles were found to be adequate.*

- Page 8: Can the chord length of the B105 rotor be provided?

Response: *Chord length has been added to the reference blade description.*

- Page 8, Left column, the last 3rd line: “as” to “is”.

Response: *Done*

- Page 8: CFD needs to be given with more detail. Is it an unsteady code?

Response: *The DLR unstructured Navier-Stokes solver TAU was used for all computations with computations performed in viscous mode on a 2D airfoil section.* *New references have been given.*

- Page 8 “Based on this fresh CFD data, unsteady aerodynamic formulation”: How are the dynamic effects (such as dynamic stall) considered?

Response: *The unsteady aerodynamic formulation is based on the Leiss model. Further description has been added to this section in the article.,
** ***

- Some references are difficult to be accessed. **--** Response: *References have been updated.*
- Results: The objective function was indicated for the inner loop. No information is given for the outer loop.

Response: *The outer loop objective function is given in Eq 5. It is the sum of the inner loop objective for each regime velocity multiplied by its weighting factor.*

- Figure 7: Why is the hinge location same for a complete mission as there are different regimes in a mission?

Response: *Physical construction of a moving hinge location is too complex and difficult to achieve. It has to be fixed for a particular blade (mission). So, in the algorithm, the hinge location is taken as the outer loop (mission) objective. Within the inner loop (regimes), the outer loop objective is kept constant.*

- Page 11, Combined chord and twist morphing: Some information about the integration is useful to be given as there are inner and outer loops in both techniques.

Response: *A short description has been added.*

- Abbreviations: Some symbols are not listed. Please complete the list.

Response: *Done*
